# Baltimore College of Dental Surgery

**Published:** 1844-06

**Authors:** 


### Miscellaneous Notices.

*Baltimore College of Dental Surgery.*—The regular Course of Lectures in this Institution begin the first Monday in November next, and continue four months.

The readers of the Journal are aware of the existence and locality of the Dental College, and of its being established and receiving its charter from the Legislature of Maryland, about four years ago.

They are also aware, that the great leading object in establishing this school, was and is, the elevation of the standard of the dental profession; of making scientific dentists, by educating young men thoroughly in all the different branches of dental knowledge, and thus sending them forth into the community fully qualified to do credit to themselves, to their profession, and by their practical skill and success, to their patients.

But, notwithstanding all this is known, there still, however, seems to be an impression among many members of the dental profession, that the Baltimore Dental College imparts to the student much more of a theoretical, than of that real substantial practical knowledge which is to constitute the main business of his life. It is to correct this error that the following remarks are principally intended.

It is true, that the principles of dental science are fully discussed, and their great value and importance seriously enforced, through the whole course, upon the attention of the student, as the great and only true guide—

the only unerring compass, by which he can either rationally, successfully, or satisfactorily regulate, and honorably conduct his after practical life.

That the principles of dental science should be taught, we do not for a moment suppose any well informed dentist hesitates a single instant to admit, and further, that these principles are the acknowledged levers—the only trustworthy and great fundamental truths, on which is reared the great structure of the dental art, and only by and through which, this art can ever expect to be practiced with becoming respect, dignity, and success.

This may be acknowledged as true, but it is still urged that there is not sufficient attention given to the surgical and mechanical parts of dentistry; in other words, it is feared, the head is educated too much, and the hand too little.

To this we reply, and it gives us great pleasure to correct so false an impression, that there is a distinct and separate chair, for teaching Surgical and Mechanical Dentistry exclusively, and that this chair is filled by a professor, who devotes his whole time to this department, and not only demonstrates to the student the different manipulations, but further requires, that he shall do for himself all the practical duties, and not only once, but this is his business to drill his fingers from day to day, through the whole course of lectures.

To enable the professor to carry out this practical plan of teaching all the students at once, the College is furnished with abundant room, and in which one is set apart expressly as a surgical and mechanical workshop, containing all the instruments and appliances suited to the different manipulations of these two practical branches, and sufficiently commodious to accommodate a large class. Ample opportunity is given to each student to witness and perform every variety of operation upon the natural or living teeth.

Here it is that surgical and mechanical dentistry is taught, and our readers will thus see that ample provision has been made for teaching the *practical* part of the profession.

The other departments comprise:

Dental Physiology and Pathology,

Special Pathology and Therapeutics, and

Anatomy and Physiology.

In each of these, it is likewise the constant aim of the several professors to make them as practical as possible. For example, anatomy is thoroughly taught on the fresh subject, and each student is likewise expected to dissect for himself. For this purpose the college contains a dissecting room, not inferior to that of any medical school, and always abundantly supplied with subjects.

The College, we may add further, has a general lecture room and a museum, which, with one of the finest dental libraries in the world—though not in the College, but to which the students can have free access—furnishes some idea of the facilities and advantages which it gives to those who come to its halls for instruction in dental science.

And it is firmly believed, and we think may be confidently asserted, that in even a single course spent in such an Institution, more real knowledge is gained, both in the principles and practice of dental surgery, than can be acquired in two years in any private office. H.

